# Retraction: *Lactobacillus paracasei* X11 ameliorates hyperuricemia and modulates gut microbiota in mice

**DOI:** 10.3389/fimmu.2024.1517021

**Published:** 2024-11-06

**Authors:** Frontiers Editorial Office

The journal retracts the 2022 article cited above.

Following publication, concerns were raised about the duplicate publication of data in the article cited above in Frontiers in Immunology and in Frontiers in Nutrition: Cao J, Bu Y, Hao H, Liu Q, Wang T, Liu Y and Yi H (2022) Effect and Potential Mechanism of Lactobacillus plantarum Q7 on Hyperuricemia *in vitro* and *in vivo*. Front. Nutr. 9:954545. doi: 10.3389/fnut.2022.954545. An investigation was conducted in accordance with Frontiers’ policies, which confirmed the concerns. Following the Committee on Publication Ethics guidelines, this article is retracted.

The authors received a communication regarding the retraction and had a chance to respond. This communication is recorded by the publisher.

